# Outer-Inner Membrane Vesicles Naturally Secreted by Gram-Negative Pathogenic Bacteria

**DOI:** 10.1371/journal.pone.0116896

**Published:** 2015-01-12

**Authors:** Carla Pérez-Cruz, Lidia Delgado, Carmen López-Iglesias, Elena Mercade

**Affiliations:** 1 Laboratori de Microbiologia, Facultat de Farmàcia, Universitat de Barcelona, Barcelona, Spain; 2 Crio-Microscòpia Electrònica, Centres Científics i Tecnològics, Universitat de Barcelona, Barcelona, Spain; University of Würzburg, GERMANY

## Abstract

Outer-inner membrane vesicles (O-IMVs) were recently described as a new type of membrane vesicle secreted by the Antarctic bacterium ***Shewanella vesiculosa*** M7T. Their formation is characterized by the protrusion of both outer and plasma membranes, which pulls cytoplasmic components into the vesicles. To demonstrate that this is not a singular phenomenon in a bacterium occurring in an extreme environment, the identification of O-IMVs in pathogenic bacteria was undertaken. With this aim, a structural study by Transmission Electron Microscopy (TEM) and Cryo-transmission electron microscopy (Cryo-TEM) was carried out, confirming that O-IMVs are also secreted by Gram-negative pathogenic bacteria such as ***Neisseria gonorrhoeae***, ***Pseudomonas aeruginosa*** PAO1 and ***Acinetobacter baumannii*** AB41, in which they represent between 0.23% and 1.2% of total vesicles produced. DNA and ATP, which are components solely found in the cell cytoplasm, were identified within membrane vesicles of these strains. The presence of DNA inside the O-IMVs produced by ***N. gonorrhoeae*** was confirmed by gold DNA immunolabeling with a specific monoclonal IgM against double-stranded DNA. A proteomic analysis of ***N. gonorrhoeae***-derived membrane vesicles identified proteins from the cytoplasm and plasma membrane. This confirmation of O-IMV extends the hitherto uniform definition of membrane vesicles in Gram-negative bacteria and explains the presence of components in membrane vesicles such as DNA, cytoplasmic and inner membrane proteins, as well as ATP, detected for the first time. The production of these O-IMVs by pathogenic Gram-negative bacteria opens up new areas of study related to their involvement in lateral gene transfer, the transfer of cytoplasmic proteins, as well as the functionality and role of ATP detected in these new vesicles.

## Introduction

Membrane vesicles (MVs) are part of a secretion-delivery system used by many Gram-negative bacteria, which allows the long-distance dissemination of bacterial products into the environment and promotes interaction with other cells**,** thus eliminating the need for bacterial contact. They are particularly involved in inter-kingdom communication, nutrient acquisition, maintenance of the biofilm structure, predation and horizontal gene transfer [1]. Furthermore, MVs from pathogenic bacteria are secreted to deliver toxic compounds directly into the host cells, enhance bacterial survival in a hostile environment and modulate host immune response. They are enriched in virulence factors such as LPS, invasins, adhesines, inmunomodulatory compounds and lytic enzymes [2–4]. Vesicles also allow interaction between prokaryotic cells. Most bacteria package antimicrobial factors into MVs, such as peptidoglycan hydrolases that cause the lysis of Gram-negative and Gram-positive bacteria, consequently killing competitors [5].

MVs are commonly described as spherical bilayered structures with an average diameter between 20 and 250 nm, produced when a small portion of the outer membrane (OM) bulges away from the cell and entraps periplasmic content. Therefore, MVs are mainly composed by LPS, periplasmic proteins, outer membrane proteins and phospholipids [6]. However, biochemical and proteomic studies have also repeatedly noticed the presence of components from the plasma membrane and cytoplasm [7]; indeed, some authors have proposed that they are constitutional components of MVs [8]. The presence of such components has been reported in many proteomic studies of pathogenic bacteria such as *Klebsiella pneumoniae*, *Acinetobacter baumannii*, *Pseudomonas aeruginosa*, *Helicobacter pylori*, *Haemophilus influenzae*, *Francisella novicida* and *Neisseria meningitidis* [8–15]. Another proteomic study performed by Lee and co-workers on *Escherichia coli* also detected cytoplasmic proteins in MVs but not some of the most abundant proteins in the bacterial cell cytoplasm, suggesting the involvement of a specific sorting mechanism [16]. Also, when the enzymatic marker for plasma membrane NADH oxidase was assayed in MVs from *P*. *aeruginosa*, its activity was detected [17].

Cytoplasmic components other than proteins are also found in MVs. The presence of DNA has been reported in vesicles from *Haemophilus influenzae*, *Yersinia pestis*, and *Shigella flexneri* [13], [18]. The DNA detected within MVs originates from multiple sources, including chromosomic DNA, plasmid DNA and DNA from bacteriophage [19]. Furthermore, MVs mediate the transfer of virulence genes between *E*. *coli* and other enteric bacteria. Antimicrobial resistance genes can also be exported via MVs, as described for *N*. *gonorrhoeae* and *A*. *baumannii* [20], [21]. This has led to the suggestion that vesicles may be involved in the exchange of genetic material among similar bacterial species and might represent a fourth method of lateral gene transfer [19]. Despite the importance of gene transfer in pathogenic bacteria via MVs, the mechanism of DNA encapsulation remains unclear [5].

In previous work, we demonstrated by TEM and Cryo-TEM that the Gram-negative bacterium *Shewanella vesiculosa* M7^T^ naturally produces a new type of outer membrane vesicle, which can entrap cytoplasmic content through a protrusion of the outer and plasma membranes. A proteomic study of MVs from this strain detected the presence of cytoplasm and plasma membrane proteins. Moreover, DNA immunogold labeling of ultrathin sections of these double-bilayered MVs showed that DNA was packaged inside, which proved that cytoplasmic content was being delivered by MV formation. These previously undescribed bacterial structures, which represent a new model of vesiculation, were termed “outer-inner membrane vesicles” (O-IMVs) [22].

The aim of the current study was to determine whether these new O-IMVs are exclusive to the Antarctic bacteria *S*. *vesiculosa* M7^T^, or are also secreted by pathogenic Gram-negative bacteria whose MVs have been repeatedly reported to contain cytoplasmic components and DNA. For this purpose, three pathogenic bacteria: *Pseudomonas aeruginosa* PAO1, *Acinetobacter baumannii* AB41 and *Neisseria gonorrhoeae* DSM15130 and their derived MVs were analyzed by TEM and Cryo-TEM, and the presence of cytoplasmic constituents such as DNA, proteins or ATP was also evaluated.

## Material and Methods

### Bacterial strains and growth conditions

*Neisseria gonorrhoeae* DMS 15130, *Pseudomonas aeruginosa* PAO1 and the clinical isolate *Acinetobacter baumannii* AB41 were employed for this study. The clinical isolate *A*. *baumannii* AB41was kindly provided by Dr. Jordi Vila from the University of Barcelona. Unless otherwise stated, for TEM studies, *Pseudomonas* PAO1 was grown in Tryticase soy agar (TSA, Oxoid) and *A*. *baumannii* in Columbia blood agar (Oxoid), and both were incubated at 37°C for 18h, while *N*. *gonorrhoeae* was grown in chocolate agar (CHOC, Oxoid) at 37°C for 48h. For membrane vesicle isolation, *Pseudomonas* PAO1 was grown in Trypticase soy broth (TSB, Oxoid) at 37°C for 5 hours, *A*. *baumannii* AB41 in Müeller-Hinton broth (MH, Oxoid) at 30°C for 15h, and both liquid cultures were incubated in an orbital shaker at 100 rpm. Membrane vesicles from *N*. *gonorrhoeae* were retrieved from confluent cultures grown in chocolate agar plates (CHOC) for 65h at 37°C.

### MV Isolation and purification from culture media

*A*. *baumannii* AB41 and *Pseudomonas* PAO1 naturally secrete MVs into media. MVs from both strains were collected from broth cultures (500 ml) in the late log phase using an adaptation of the method described by McBroom and coworkers [23]. The cells were pelleted by centrifugation at 10,000 × *g* for 10 min at 4°C, and the supernatant was filtered through 0.45-μm-pore-size filters to remove remaining bacterial cells. MVs were obtained by centrifugation at 40,000 × *g* for 1 h at 4°C in an Avanti J-20 XP centrifuge (Beckman Coulter, Inc.). Pelleted vesicles were resuspended in 50 ml of 50 mM HEPES pH 6.8 (Sigma) and filtered through 0.22-μm-pore-size Ultrafree spin filters (Millipore). Vesicles were again pelleted and finally resuspended in an adequate volume of 50 mM HEPES, pH 6.8 (Sigma). MVs from *N*. *gonorrhoeae* were collected from confluent solid cultures grown on CHOC plates. Cells and MVs from 20 agar plates were resuspended in 15 ml of Ringer ¼ (Sigma) per plate and from this moment the MVs were obtained as described for liquid media cultures.

For proteomic studies, MVs from *N*. *gonorrhoeae* were further purified by ultracentrifugation in OptiPrep gradients as described by Pérez-Cruz et al. [22].

### TEM observation after High-Pressure Freezing and Freeze Substitution (HPF-FS)

TEM observations of the three strains and their isolated MVs were performed as described by Pérez-Cruz et al [22]. Briefly, bacterial colonies were cryoimmobilized using a Leica EMPact high-pressure freezer (Leica, Vienna, Austria). Frozen samples were freeze-substituted in a Leica EM automatic freeze substitution (AFS) system (Leica, Vienna, Austria), where the substitution was performed in pure acetone containing 2% (wt/vol) osmium tetroxide and 0.1% (wt/vol) uranyl acetate at −90°C for 72 h. The temperature was gradually increased (5°C/h) to 4°C, held constant for 2 h, and then finally increased to room temperature and maintained for 1 h. Samples were washed for 1 h in acetone at room temperature and infiltrated in a graded series of Epon-acetone mixtures, and pure Epon 812 (Ted Pella, Inc.) for 30 h. Samples were embedded in fresh Epon and polymerized at 60°C for 48 h. Epon-embedded thin sections were examined in a Tecnai Spirit electron microscope (FEI Company, Netherlands) at an acceleration voltage of 120 kV.

### Cryo-TEM observation of pathogenic bacteria cultures and their isolated MVs

Bacterial suspensions containing MVs from *N*. *gonorrhoeae*, *Pseudomonas* PAO1 and *A*. *baumannii* AB41 were visualized by Cryo-TEM. *Pseudomonas* PAO1 and *A*. *baumannii* AB41 were incubated overnight in 50 mL of TSB medium. For *N*. *gonorrhoeae*, confluent CHOC agar cultures were incubated for 24 hours and resuspended in 50 ml in PBS. For all strains, a large part of the cells were gently removed by centrifugation at 3,000 x g for 10 minutes. The remaining cells and MVs from each strain were sedimented from the supernatants by high-speed centrifugation at 40,000 x g for 30 minutes in an Avanti J-20 XP centrifuge (Beckman Coulter, Inc). The pellet was washed with 50 ml of 50 mM HEPES pH 6.8 and centrifuged again at 40,000 x g. Finally, the pellet was resuspended in an adequate volume of 50 mM HEPES pH 6.8 for Cryo-TEM observation. For that purpose, the suspension was adjusted to a turbidity equivalent to 1 McFarland standard. For Cryo-TEM observation of isolated MVs without cells, samples were prepared as described in the above method for MV Isolation and purification from culture media. In all Cryo-TEM analysis, five microliters of the corresponding suspension were applied on freshly glow-discharged Quantifoil R 2/2 grids (Quantifoil Micro Tools GmbH, Jena, Germany). The sample was allowed to adhere to the grids for 1 minute. The excess of liquid was then blotted with filter paper, leaving the sample films spanning the grid holes. The samples were vitrified by plunging the grid into ethane, which was kept at melting point with nitrogen using a Vitroblot (FEI Company, Eindhoven, Netherlands), and maintained before freezing at 100% humidity and room temperature. The vitreous sample films were transferred to a Tecnai F20 microscope (FEI Company, Eindhoven, Netherlands), using a Gatan cryotransfer system (Gatan Inc. Pleasanton, CA). Cryo-TEM visualizations were carried out at a temperature between −170°C and −175°C and at the accelerating voltage of 200kV. Images were acquired using low-dose imaging conditions and an Eagle 4k x 4k Images charged-coupled device (CCD) camera (FEI Company, Eindhoven, Netherlands). The different type of vesicles and the diameters were analyzed using Image J software [24]. Each experiment was performed in duplicate.

### Proteomic analysis of MVs from *N*. *gonorrhoeae*

A proteomic analysis of purified MVs from *N*. *gonorrhoeae* was carried out using one-dimensional (1-D) SDS-PAGE and nano-liquid chromatography-tandem mass spectrometry (LC-MS/MS) analysis as described by Pérez-Cruz et al. with some modifications [22]. Briefly, proteins were in-gel digested with trypsin, and the tryptic peptides were extracted from the gel matrix, pooled, and dried in a vacuum centrifuge. The dried-down peptide mixture was analyzed in a nanoAcquity liquid chromatographer (Waters) coupled to an LTQ-Orbitrap Velos (Thermo Scientific) mass spectrometer. A database was created merging all Uniprot-SwissProt and Uniprot-TrEMBL entries for *Neisseria gonorrhoeae* with Uniprot-Swissprot all (September 2013). The software Thermo Proteome Discover (v.1.3.0.339) was used to perform a search by the Sequest search engine against this database. The results were filtered and only proteins identified with at least 2 high confidence peptides (FDR≤ 0.01) were included. Bacterial protein subcellular localization was predicted with the software PSORTb v3.0.2 [25].

### Fluorometric DNA quantification

Surface-associated DNA and DNA contained within MVs were quantified by the PicoGreen assay (Molecular Probes) as described by Pérez-Cruz et al. [22]. MVs were collected from three independent experiments. For each experiment, two 30-μg protein samples from MVs were prepared. One was pretreated with 50 μg/ml pancreatic DNase I (Sigma) and 10mM MgCl_2_ (1 h at 37°C) to digest DNA located outside the OMVs. Both MVs samples were lysed with 0.125% Triton X-100 solution. Samples were further processed according to the manufacturer’s instructions, and fluorescence was measured in a Modulus Microplate Multimode Reader (Turner Biosystems). Each experiment was performed in triplicate and the results were expressed as mean ± standard deviation (SD).

### DNA Immunolabeling of MVs from *N*. *gonorrhoeae* thin sections

HPF-FS of isolated MVs from *N*. *gonorrhoeae* was carried out as described previously by Pérez-Cruz et al. [22]. Colloidal gold immunolabeling of Lowicryl-HM20 thin sections was carried out as follows. Grids were pre-treated with a solution of 100 μg/ml proteinase K (Sigma) to expose the DNA before the immunolabeling. Unless specified, washing steps were carried out by floating the grids face down on the drops. Grids with sections were floated on 0.1 M PBS for 3 min. The grids were blocked on 0.1 M PBS–50 mM glycine and rinsed with 0.1 M PBS, again with 2.5% BSA–0.1 M PBS (1 drop for 3 min and 1 drop for 12 min), and again with 1% BSA–0.1 M PBS (1 drop for 8 s). Next, the grids were incubated with monoclonal IgM specific for double-stranded DNA (dsDNA) (clone AC-30–10; Novus Biologicals), diluted 1/2 in 0.1% BSA–0.1 M PBS, for 1 h. Grids were washed for 5 min on 5 drops of 0.25% Tween 20–0.1 M PBS, followed by 3 min on 1 drop of 0.1% BSA–0.1 M PBS. After that, grids were incubated for 30 min with a secondary goat anti-mouse antibody coupled to 12-nm colloidal gold (lot 84359; Jackson) diluted 1/20 in 0.1% BSA–0.1 M PBS. Grids were washed with 0.1 M PBS, followed by double-distilled water and then floated on 1% glutaraldehyde–0.1 M PBS for 5 min. Grids were rinsed with double-distilled water and dried with filter paper. Finally, grids were post-stained with 2% uranyl acetate–methanol for 5 min, 70% methanol for 3 min, 50% methanol for 3 min, and 30% methanol for 3 min, jet-washed with double-distilled water, air dried for 20 min, stained with lead citrate for 2 min, and jet-washed with double-distilled water. Two controls were used. First, the dsDNA monoclonal antibody was omitted. Second, the grids were preincubated at 37°C for 16 h with 1 mg/ml DNase I (Sigma) in PBS plus 7 mM MgCl_2_, and then the grids were thoroughly washed with water before immunolabeling with the dsDNA monoclonal antibody.

### Luminescent quantification of ATP

ATP contained in MVs was measured by a BacTiter-Glo Microbial Cell Viability Assay (Promega). Isolated MVs were retrieved from exponential growing cultures as described above and samples were stored at −80°C to avoid ATP degradation. Aliquots of each MV preparation from the three assayed strains were inoculated into the corresponding fresh culture media and incubated overnight at 37°C to check for bacterial contamination. 100 μl of isolated MVs were processed according to the manufacturer’s instructions and the luminescence was measured in Modulus Microplate Multimode Reader (Turner Biosystems). The standard curve was prepared from 1 μM of Adenosine 5’-triphosphate sodium salt (Promega) initial solution and 10-fold serial dilutions were done (1 μM to 10 pM). ATP concentrations in MV samples were determined by comparing the signal emitted with the ATP standard curve for each assay. The protein concentration of each sample was determined. Each experiment was performed in duplicate and the ATP content was normalized by protein concentration.

## Results

### TEM observation of O-IMVs in pathogenic bacteria after HPF-FS

We performed a structural analysis by TEM to determine if three pathogenic bacteria, *N*. *gonorrhoeae*, *Pseudomonas* PAO1 and *A*. *baumannii* AB41, were able to secrete different types of membrane vesicles in the same way as we had previously demonstrated in the non-pathogenic Antarctic bacteria *Shewanella vesiculosa* M7^T^ [22]. From now on, membrane vesicles (MVs) will refer to all the vesicles isolated from one bacterium, outer membrane vesicles (OMVs) are the conventional single bilayered membrane vesicles, and finally, O-IMVs refer to the new double-bilayer membrane vesicles.

**Figure 1:**
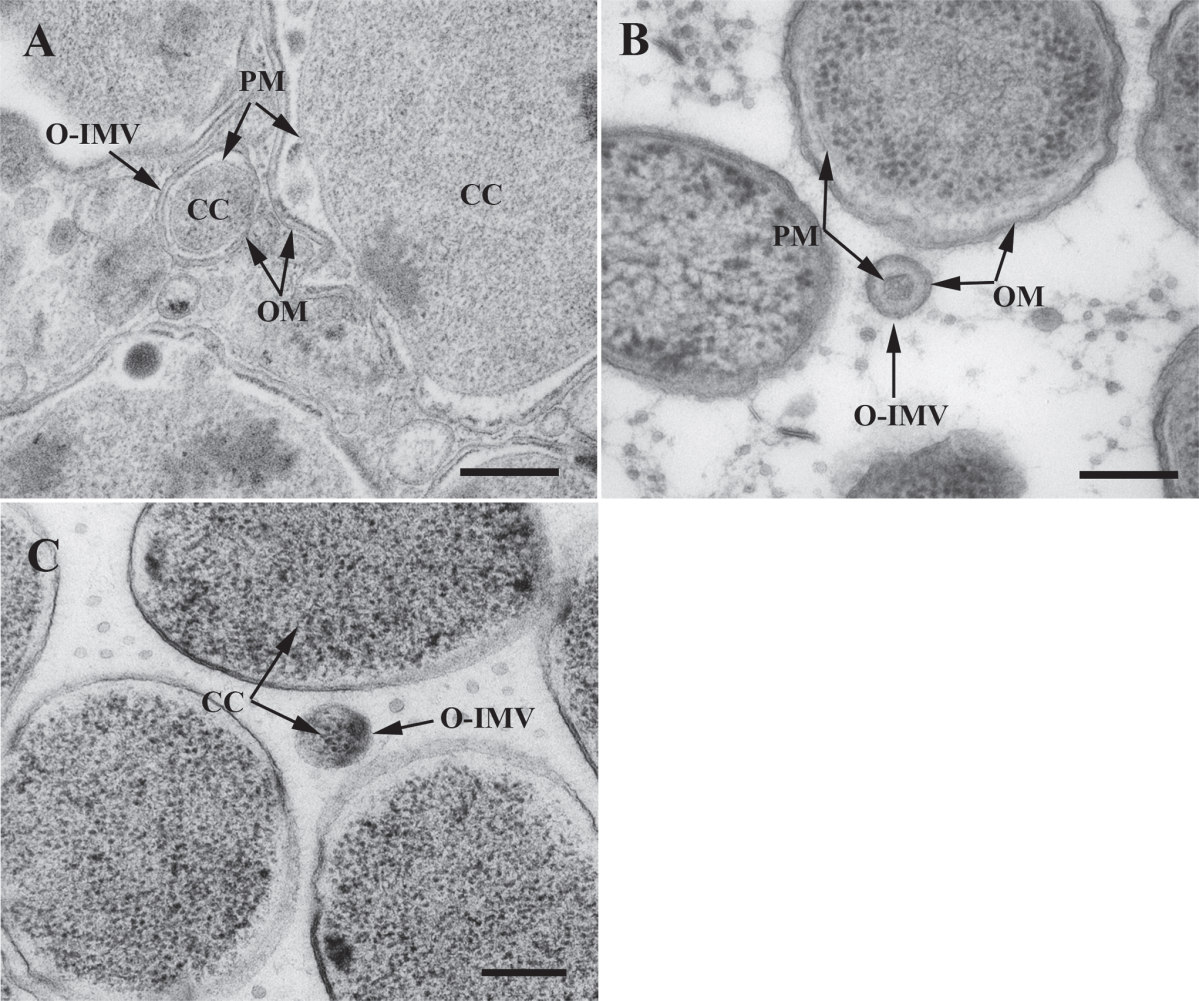
MVs visualized by TEM in pathogenic bacteria. HPF-FS sections correspond to the three pathogenic strains grown in solid media: (A) *N*. *gonorrhoeae*, (B) *Pseudomonas* PAO1 and (C) *A*. *baumannii*. O-IMVs (marked with arrows) are observed in the extracellular matter of the three pathogenic bacteria. O-IMVs in A and B clearly show a double bilayer, which exhibits the same staining profile as OM and PM from the respective cells. The O-IMV inner membrane encloses a material similar to that seen in the cytoplasm of the respective cells. OM: outer membrane; PM: plasma membrane; CC: cytoplasmic content. Bars 200 nm.

**Figure 2:**
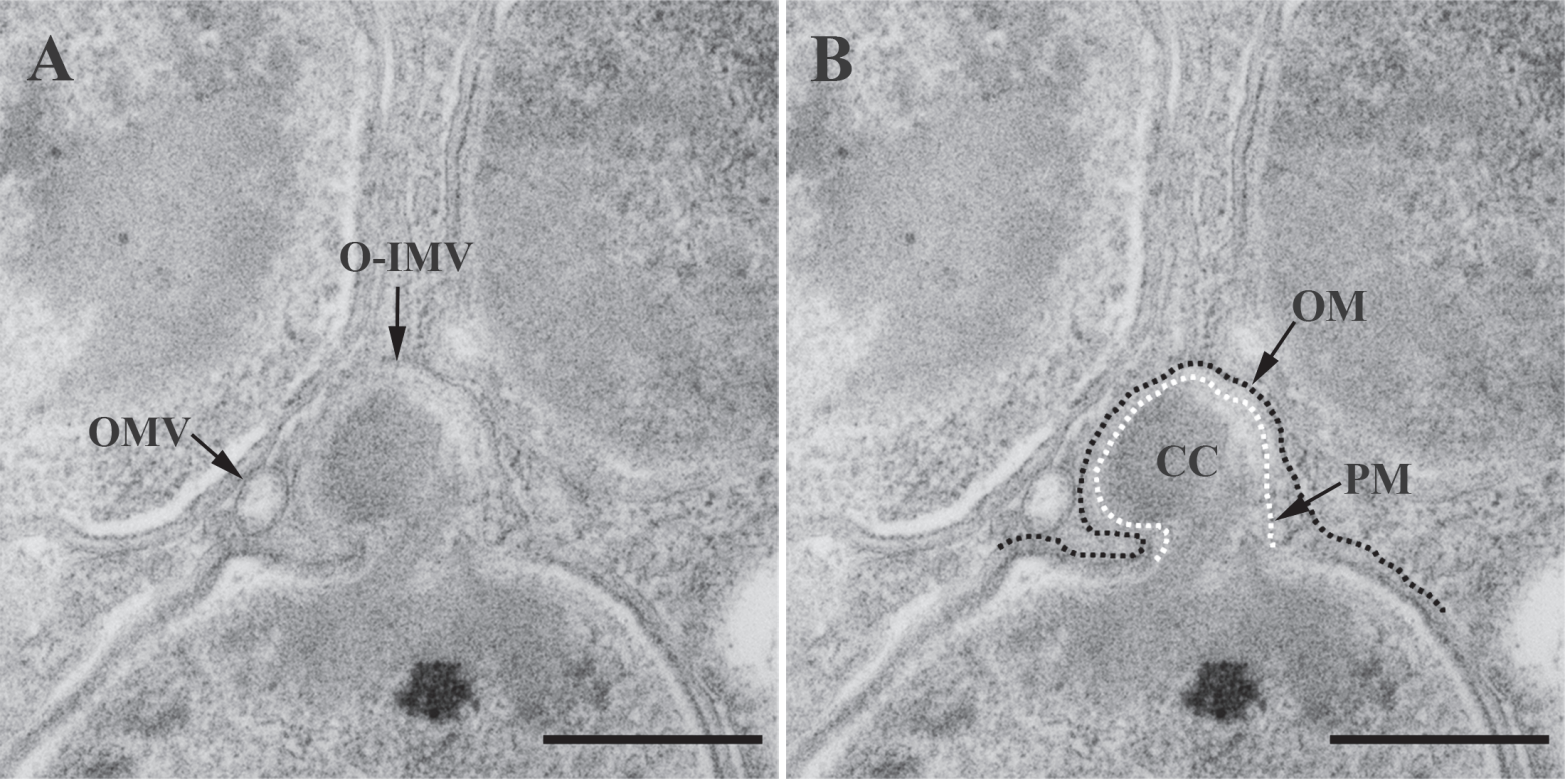
An O-IMV being released from the surface of a *N*. *gonorrhoeae* cell. (A) The TEM micrograph provides a view of an O-IMV at the moment of its formation, where the outer membrane (OM) is being extruded, dragging along the plasma membrane (PM) and a portion of the cytoplasmic content (CC). (B) The same image as A but with the cell envelope outlined to highlight the formation and structure of the O-IMV. Bars, 200 nm.

TEM observations of ultrathin sections of whole cells obtained from solid cultures after HPF-FS revealed extracellular matter containing large amounts of MVs. The conventional OMVs with a single bilayer were predominant, exhibiting the same structure, width, and staining profile as the outer cell membrane (OM). Less frequently, in sections from each of the three analyzed bacteria, O-IMVs with a double bilayer were also visualized (Fig. 1). Inside the inner membrane of the O-IMVs we observed a highly electron-dense material, similar to that seen in the cell cytoplasm, and small rough areas resembling cytoplasmic ribosomes. Furthermore, in *N*. *gonorrhoeae* we clearly observed an O-IMV precisely at the moment of formation (Fig. 2), which confirmed that the external membrane derived from the cell OM, and the inner membrane corresponded to the cell plasma membrane (PM). It should be stressed that these O-IMVs were naturally secreted without the presence of any disturbing factor (e.g antibiotic, chelating agents or antibodies), and appeared to pinch off from cells that seemed otherwise undamaged and featured intact cell envelopes.

**Figure 3:**
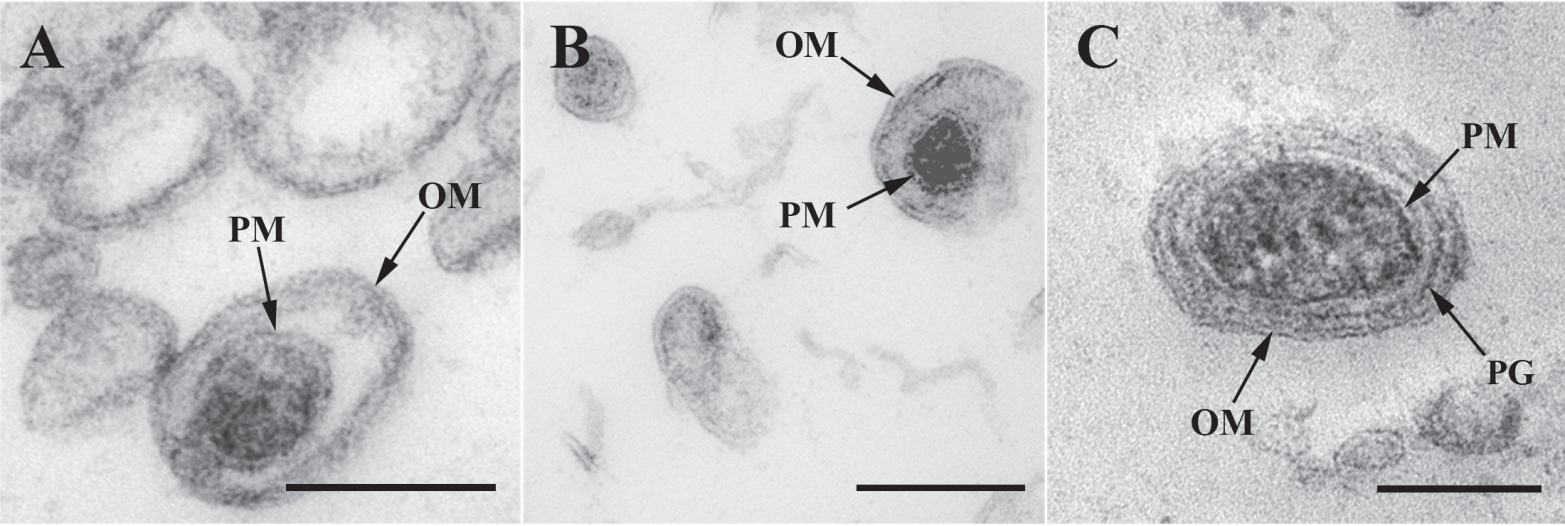
O-IMV visualized by TEM. TEM micrographs from HPF-FS sections of MVs isolated from (A) *N*. *gonorrhoeae*, (B) *Pseudomonas* PAO1 and (C) *A*. *baumannii*. O-IMVs observed in MV preparations from the three strains have certain features in common: all are surrounded by an external bilayer, probably corresponding to the outer membrane (OM) of the cell, and contain an inner membrane, probably corresponding to the plasma membrane (PM) of the cell, which entraps a high electron-dense material. In the image of O-IMVs from *A*. *baumannii* the putative peptidoglican layer (PG) can be seen. Bars 100 nm.

For the same purpose, MVs from the three strains grown on liquid cultures were isolated and analyzed by TEM after HPF-FS (Fig. 3). Although single-bilayer OMVs predominated in all observed fields, the new O-IMVs were detected in each of the three pathogenic bacteria, and presented a similar structure, which included a double bilayer surrounding highly electron-dense material.

### Cryo-TEM analysis of pathogenic bacteria and O-IMV observation

To confirm the existence of the new O-IMVs in the three analyzed pathogenic strains, accurate ultrastructural image data of the bacterial cells and their associated MVs was obtained by Cryo-TEM in a close-to-native state. Bacterial cell suspensions were obtained from exponentially growing cultures of *Pseudomonas* PAO1 and *A*. *baumannii*, and from confluent solid cultures of *N*. *gonorrhoeae*. The suspensions were subjected to gentle centrifugation to eliminate the majority of the cells. The clarified supernatants were then centrifuged at high speed to sediment the remaining cells and their MVs. The pellet was resuspended, vitrified and imaged by Cryo-TEM at −178°C using the low-radiation-dose scheme.

**Figure 4:**
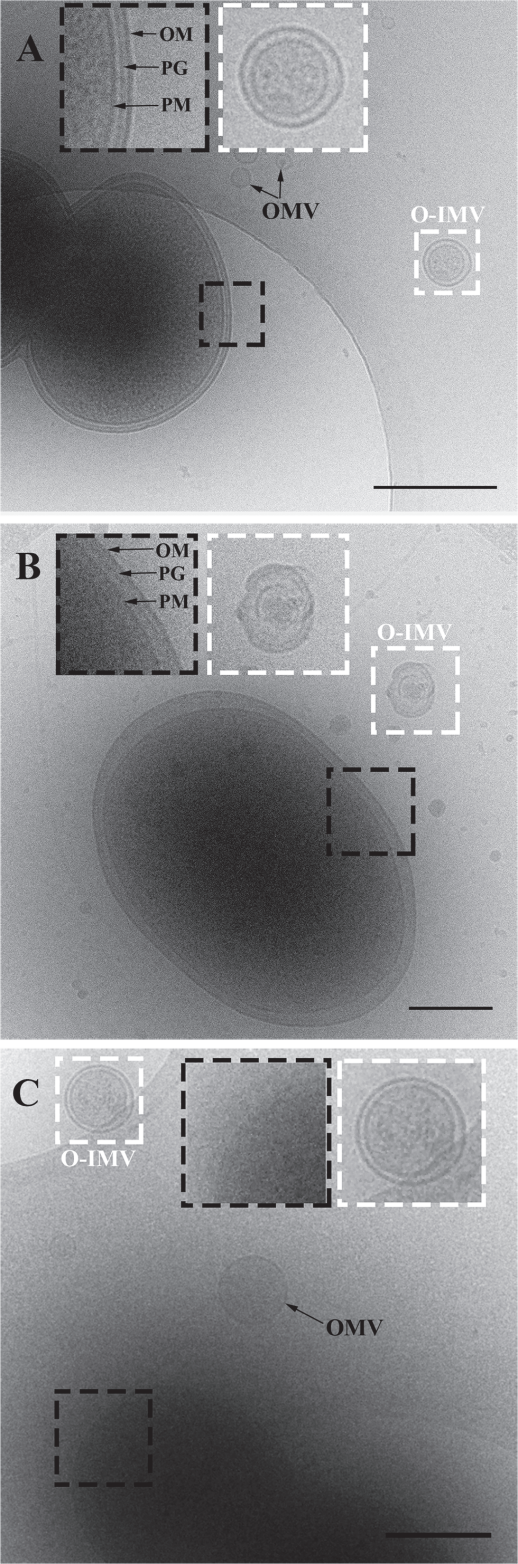
Cryo-TEM visualization of O-IMVs in pathogenic bacteria. Cryo-electron micrographs showing whole plunge-frozen cells from three pathogenic bacteria, and their derived O-IMVs: (A) *N*. *gonorrhoeae*, (B) *Pseudomonas* PAO1, and (C) *A*. *baumannii*. Whole cells with well-defined envelopes are observed in A and B (large black squares show a magnified area of cell envelopes). The new O-IMVs in the three analyzed samples exhibit the same double layer as cells, and are filled with an electron-dense material similar to that seen in the cell cytoplasm (large white squares show a magnified area of the O-IMV). Conventional OMVs are also visualized in images A and C (black arrows). OM: Outer Membrane; PM: Plasma membrane; PG: Peptidoglycan. Bars, 500 nm (A, C) and 250 nm (B). ****

The major features of the cell envelopes of the Gram-negative bacteria, *N*. *gonorrhoeae*, *Pseudomonas* PAO1 and *A*. *baumannii* AB41, were visualized by Cryo-TEM. Since plunge-frozen cells are not cryo-sectioned as in CEMOVIS but are observed whole, their thickness only allows a good resolution of the outer part of the cell. Outer and plasma membranes, separated by the periplasmic space in which the peptidoglycan layer was identifiable, were observed (Fig. 4A and B, black squares). The bilayer structure of the outer and plasma membranes was visualized in few cases. Cell membranes from the three strains had a smooth appearance, without any protuberances that might suggest vesiculation. Secreted MVs were observed in all preparations (Fig. 4). The conventional OMVs showed the typical single-layer structure and were filled with a low electron-dense material. In contrast, the new O-IMVs showed two layers with the same profile as the cell OM and PM, entrapping a highly electron-dense material similar to the cytoplasmic content of the cell (Fig. 4, white squares). The sample thickness of whole-mounted plunge-frozen cells (more than 500 nm) made it difficult to simultaneously focus both the cells and the small vesicles. This was especially the case for small conventional OMVs (about 20 nm), which were hardly observed despite their abundance. In all cryo-fixed samples, cells presented a well-preserved ultrastructure and no cellular remnants or signs of cellular stress and cell disintegration were visible.

### Quantification of O-IMVs from pathogenic strains by Cryo-TEM

To determine the frequency at which O-IMVs were produced in different pathogenic strains, and to obtain accurate measurements of the diameters of each type of vesicle, we analyzed whole plunge-frozen MV samples by Cryo-TEM. MVs from *N*. *gonorrhoeae* were obtained from confluent cultures in solid agar, while *Pseudomonas* PAO1 and *A*. *baumannii* AB4 MVs were retrieved from exponentially growing cultures. The growth conditions assayed for all the strains were designed to avoid the presence of circularized membrane fragments from cell debris. The vesicles were obtained from cell-free supernatants according to a standard high-speed centrifugation protocol that ensures the recovery of even the smallest vesicles.

**Figure 5:**
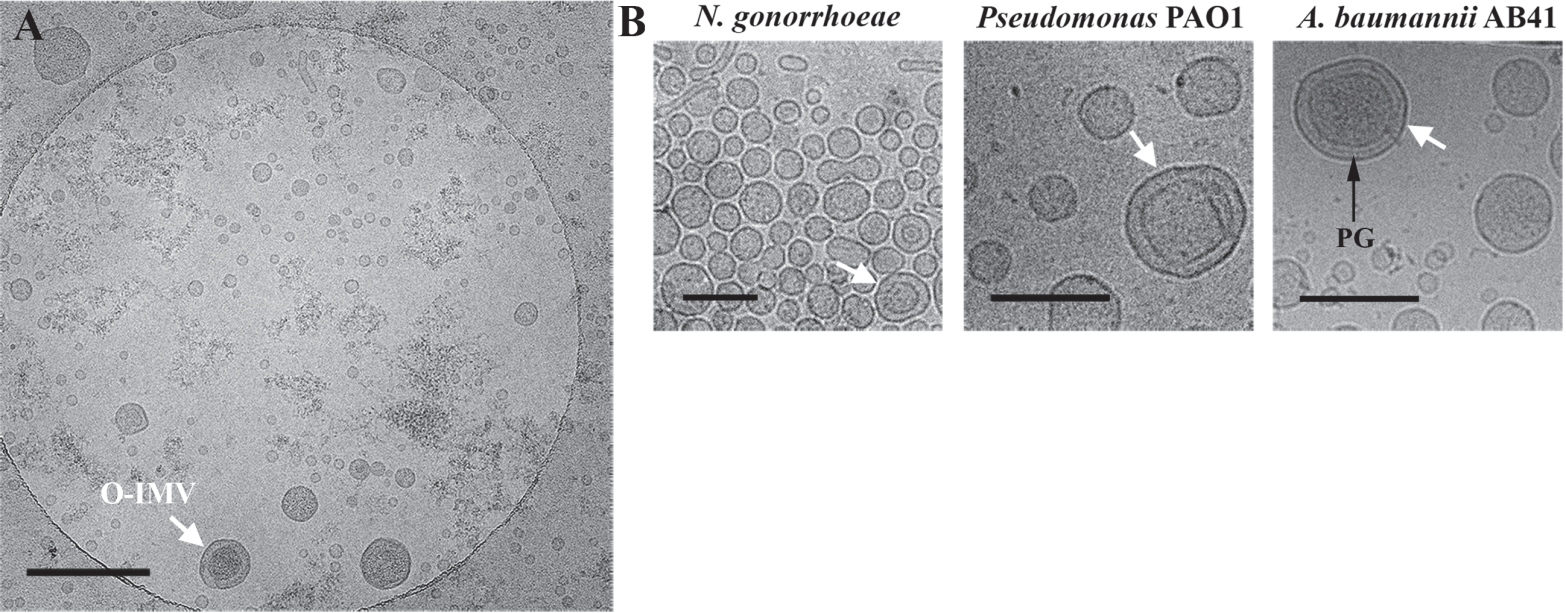
Quantification of O-IMVs on thin frozen foils from the total MVs using Cryo-TEM. (A) Overview of a thin frozen foil obtained from *A*. *baumannii*. *S*ingle-layer vesicles are highly abundant, while double-layer O-IMVs can be observed in all the tracked fields, but much less often (white arrows). (B) Cryo-TEM images of thin frozen foils from the MVs of the three assayed strains. Both types of vesicles are observed, the single-layer OMVs and the new double-layer O-IMVs (white arrows). In *A*. *baumannii* O-IMVs, the presence of the putative intact peptidoglycan layer is also observed (PG). Bars 500 nm (A) and 200 nm (B).

As in the extracellular matter from the three assayed strains, O-IMVs were also visualized by Cryo-TEM (Fig. 5, white arrows), although single-layer OMVs predominated in all analyzed fields. After counting more than 7000 vesicles per strain in two independent experiments, the percentage of O-IMVs with respect to the total MVs was 0.54% ± 0.058 for *Pseudomonas* PAO1, 0.23% ± 0.056 for *A*. *baumannii* AB41, and 1.2% ± 0.07 for *N*. *gonorrhoeae*. Although the proportion of O-IMVs observed in *N*. *gonorrhoeae* was notably higher, it does not mean this strain produces more of this new type of vesicle, because the vesicles were obtained from confluent solid cultures and not from liquid cultures, rendering the results incomparable.

The mean diameter and diameter range were also measured for both types of MVs (Table 1). Although the range of diameters was very broad for each type of vesicle and for each strain, most OMVs presented a diameter of 40–60 nm for *N*. *gonorrhoeae*, 20–40 nm for *A*. *baumannii* and 50–100 nm for *Pseudomonas* PAO1, while the new O-IMV vesicles were larger, mostly between 60–100 nm for *N*. *gonorrhoeae*, 100–140 nm for *Pseudomonas* PAO1 and 125–160 nm for *A*. *baumannii*. No membrane fragments from lysed cells were observed in thin frozen foils of MVs from *N*. *gonorrhoeae* and *A*. *baumannii*, while only a few were detected in *P*. *aeruginosa* PAO1 MV preparations.

**Table 1:** Diameter mean values and diameter ranges expressed in nm from OMVs and O-IMVs for each analyzed strain.

Strain	OMV		O-IMV	
	Mean ± SD	Range	Mean ± SD	Range
N. gonorrhoeae	57± 19	25–140	85 ±26	40–170
*Pseudomonas* PAO1	85 ± 28	30–165	135 ± 34	55–145
*A*. *baumannii* AB41	44 ± 32	15–320	109 ± 52	65–260

### DNA content in MVs from pathogenic bacteria

MVs isolated from exponentially growing cultures were used to measure the DNA content before and after a DNase treatment. The DNA was quantified by the Picogreen assay, which is an ultrasensitive method for the detection of double-stranded DNA, with minimal fluorescence contributed by RNA and single-stranded DNA. The DNA content of MVs from the three analyzed strains can be seen in Table 2. As expected, the DNA quantified was encapsulated mostly inside the MVs and protected from hydrolysis by exogenous DNases.

**Table 2:** DNA and ATP quantification in MVs.

Strain	DNA^a^			ATP ^b^
	(ng DNA / μg prot)			(nmol/g protein)
	DNase	No DNase	% DNase protected	
N. gonorrhoeae	1.11 ± 0.26	1.87 ± 0.42	59	0.57 ± 0.27
*Pseudomonas* PAO1	0.13 ± 0.04	0.20 ± 0.06	65	0.09 ± 0.01
*A*. *baumannii* AB41	1.54 ± 0.30	2.64 ± 1. 23	62	2.61 ± 1.07

Data are the means ± standard deviations of the average intensity from: (a) three independent assays, (b) two independent assays.

### Immunolabeling reveals DNA is packaged inside O-IMVs

**Figure 6:**
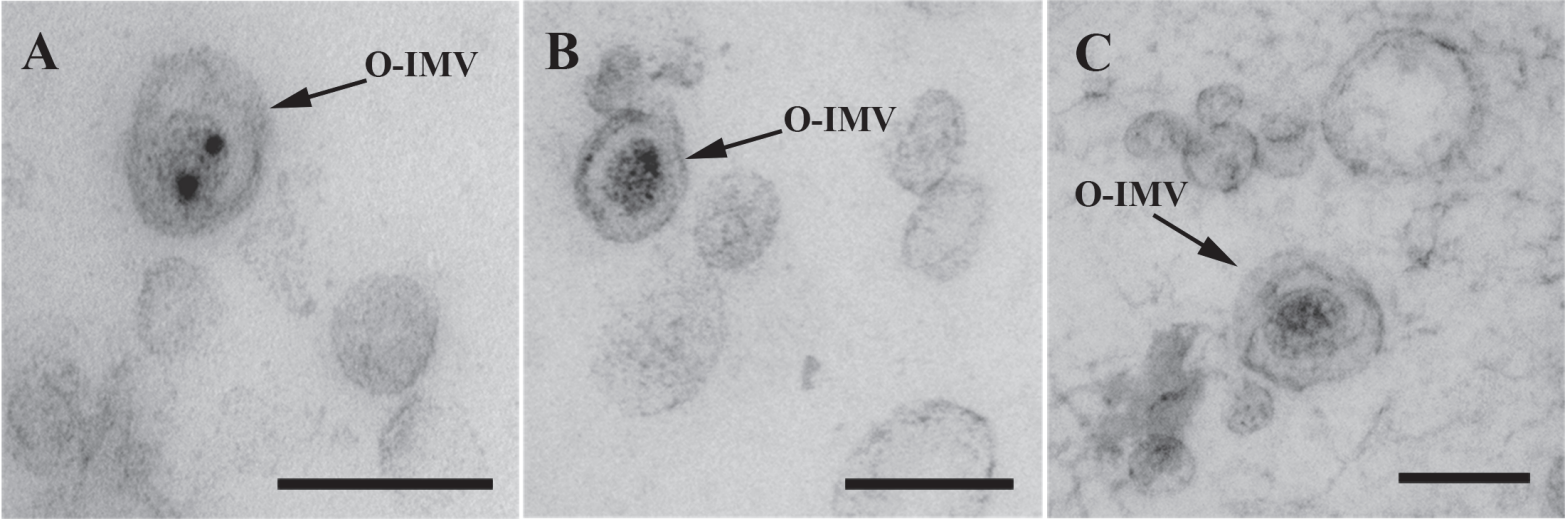
DNA gold immunolabeling on Lowicryl HM20 thin sections of HPF-FS isolated MVs from *N*. *gonorrhoeae*. (A) TEM micrograph showing an O-IMV immunolabeled with a monoclonal IgM specific against dsDNA and a secondary goat anti-mouse antibody coupled to 12-nm colloidal gold. The gold mark is localized inside the inner layer that contains the electron-dense material, which confirms that the DNA is packaged within the new O-IMV. (B) TEM micrograph of MVs labeled only with the secondary antibody. (C) TEM micrograph of MVs from grids preincubated with 1 mg/ml DNase I and then immunolabeled with the anti-dsDNA IgM and a secondary antibody coupled to gold. Bars, 100 nm.

To verify that DNA was present in the new O-IMVs and not in conventional OMVs, we performed an immunogold labeling of MVs with an antibody specific for double-stranded DNA. We used the vesicles isolated from *N*. *gonorrhoeae*, since this strain secreted a larger amount of O-IMVs than the other assayed bacteria. For this assay, thin sections of MV samples were deposited onto grids. Before the immunolabeling, the grids were digested with 100 μg/ml of proteinase K for 15 minutes at 37°C to expose the DNA. Both conventional OMVs with a single-bilayer and the double-bilayer O-IMVs were observed (Fig. 6), and as expected, the gold mark appeared almost exclusively in the latter, specifically inside the inner membrane of vesicles containing an electron-dense material (Figs. 6A and S1). To check that the gold immunolabeling was specific, we conducted several control experiments. No gold marking was observed when the primary antibody was not used (Fig. 6B) or when the grids were pretreated with DNase before the immunolabeling (Fig. 6C), showing that the DNA within double-bilayer vesicles was degraded by DNase treatment.

### Cytoplasm and plasma membrane components detected in *N*. *gonorrhoeae* MVs by proteomic analysis

**Figure 7:**
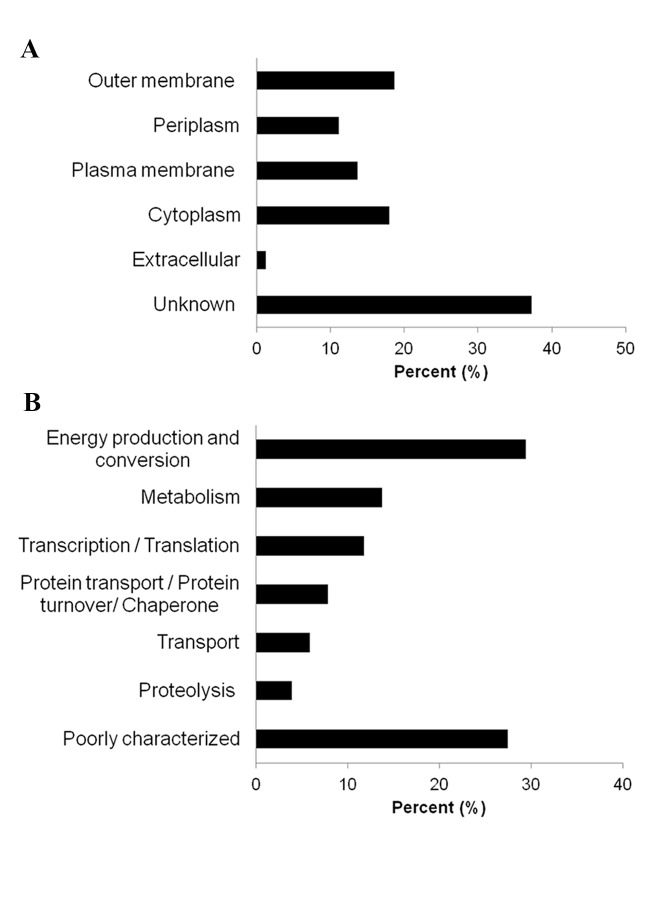
Protein content from the *N*. *gonorrhoeae*-derived MVs. (A) Distribution of proteins identified from the total MVs from *N*. *gonorrhoeae* based on their subcellular location. (B) Functional classification of the 51 proteins predicted to be localized in the cytoplasm and plasma membrane of *N*. *gonorrhoeae* cells.

Previous proteomic studies of MVs isolated from *P*. *aeruginosa* and *A*. *baumannii* reported the presence of cytoplasmic and PM proteins, although the mechanism by which they are incorporated in the vesicles has remained perplexing [9], [10]. We carried out a proteomic analysis of MVs from *N*. *gonorrhoeae*, which has not been described previously, to identify the proteins associated with vesicles in this pathogenic bacterium and determine their subcellular localization. Isolated MVs were further purified on an OptiPrep density gradient to remove contaminants. The 161 proteins detected in the MVs from *N*. *gonorrhoeae* were classified according to their cellular localization, which was established using the bacterial localization prediction software PSORTb v3.0.2 (Fig. 7A). OM proteins constituted 18.6% of all proteins identified, and were mainly associated with the cell wall and membrane biogenesis and transport. Notably, some of these OM proteins identified in *N*. *gonorrhoeae* vesicles are known to act as gonococcal virulence factors, including Pilus-related proteins, opacity-associated (Opa) OM proteins, and the complement regulatory *Neisseria* surface protein (NspA). Periplasmic proteins, mainly related with nutrient transport, were also identified (11.2%). As in nearly all proteomic studies of bacterial MVs, the proteomic analysis of MVs from *N*. *gonorrhoeae* identified proteins from the PM (13.7%) and the cytoplasm (18%), whose broad range of functions are summarized in Fig. 7B. The most abundant proteins with a known function localized in the PM were a carboxy-terminal processing protease and the thiol:disulfide interchange protein DsbD involved in the cytochrome complex assembly. The most abundant proteins identified in the cytoplasm were a pyruvate dehydrogenase, the 60 kDa chaperonin and the elongation factor Tu. Similar to the proteome profiles of other bacterial MVs, we found ribosomal proteins, specifically 50S ribosomal protein L1 and the 30S ribosomal proteins S7 and S9. Few extracellular proteins were identified (1.2%), and many proteins with unknown subcellular localization or multiple localization sites were also detected (37.3%), some of them being virulence factors, such as the IgA1 endopeptidase and proteins from the PilS cassette. The subcellular localization, biological function and molecular function of each protein identified in MVs from *N*. *gonorrhoeae* are available in the Supporting Information (S1 Table).

### ATP detection in MVs from pathogenic bacteria

ATP is a cytoplasmic component and is the universal energy currency used in many biological processes. In recent years, several reports have detected the presence of extracellular ATP in the culture supernatants of a wide variety of Gram-positive and Gram-negative bacteria, but the mechanism for its secretion is not clear [26]. We performed an ATP quantification in MVs isolated from the three pathogenic strains using the BacTiter-Glo Microbial Cell Viability Assay. This luciferase-based assay correlates the luminescence emitted with an ATP standard curve. ATP was detected inside the MVs of each pathogenic strain, and the luminescent signal obtained for all samples was about 10^5^ Relative light units (RLU). The ATP concentration referred to protein is detailed in Table 2.

## Discussion

The existence of membrane vesicles (MVs) was first discovered by use of electron microscopy in the 1960s in culture supernatants of *Escherichia coli* [1]. Since then, the production of vesicles with multiple functions has been widely observed in both prokaryotic and eukaryotic organisms [27]. In Gram-negative bacteria, these vesicles have been named “outer membrane vesicles” (OMVs), and are known to favour virulence and host colonization in many pathogenic bacteria [2].

Although knowledge of OMV functions in bacteria has increased substantially in recent years, the mechanism by which vesiculation occurs remains unclear. Current models of vesiculation, which begin with budding events observable by electron microscopy, all coincide that OMVs can be defined as small spherical structures of 20–200 nm derived from the bacterial OM and that they have a common structure comprising an outer lipid bilayer surrounding material from the periplasm [1], [27]. This definition of OMVs has remained unchanged since their discovery, suggesting that Gram-negative bacteria produce a single morphological type of vesicle. The repeated detection of cytoplasmic components and plasma membrane proteins, which in accordance with this definition should not be present in the vesicles, has not been explained [5], [7], [18], [27].

In recent years, improvements in TEM and Cryo-TEM techniques have enabled the imaging of biological specimens with greatly enhanced resolution. TEM observation of specimens cryoimmobilized by High Pressure Freezing (HPF) followed by Freeze Substitution (FS) and sectioning, together with Cryo-TEM observation of frozen-hydrated specimens, allow visualization of biological samples close to their native state, enabling us to refine our knowledge of known bacterial structures and to discover new ones [22], [28], [29]. These techniques allowed us to visualize the formation of a new type of MVs in the Antarctic bacterium *Shewanella vesiculosa* M7^T^ [22].

The structural study of the three pathogenic strains by TEM showed extracellular matrices full of MVs, which were mainly conventional OMVs. However, also visualized in several of the analyzed fields were the new O-IMVs, with a double-bilayer and containing highly electron-dense material very similar to cytoplasmic content. In addition, small rough areas, very similar to the ribosomes found in the cell cytoplasm, were also observed in some O-IMVs.

Although analysis of ultrathin sections by TEM after HPF-FS currently allows a good preservation of cell components and also correctly displays the extracellular matrices, this process is not completely free of artifacts [22], [29]. For this reason, the three strains were also analyzed by Cryo-TEM, because this technique is the least artifactual and allows the sample to be visualized close to the natural state, with minimal sample manipulation. For visualization by Cryo-TEM, bacteria are embedded in a thin layer of water, vitrified by rapid immersion in ethane and directly visualized by Cryo-microscopy at liquid nitrogen temperatures without any added contrast agent [30].

Through Cryo-TEM, we were again able to demonstrate that all three pathogenic strains produce both types of MVs: single layer OMVs and double layer O-IMVs containing a material similar to the bacterial cytoplasm. Thus, structural analysis by TEM and Cryo-TEM confirmed that *N*. *gonorrhoeae*, *P*. *aeruginosa* PAO1 and *A*. *baumannii* AB41 produce more than one type of MV, showing a predominance of OMVs and a small proportion of the new O-IMVs.

The reason why these new vesicles have not been visualized before is probably their low rate of production. O-IMVs constituted close to 0.2% of all vesicles observed in *A*. *baumannii* AB41, about 0.5% in *P*. *aeruginosa* PAO1 and 1.2%, in *N*. *gonorrhoeae*. Consequently, their detection by TEM and Cryo-TEM required the analysis of many fields. These low proportions of O-IMVs seem reasonable when considering that their formation involves momentary ruptures of the cell wall and plasma membrane, which may compromise cell viability. This suggests their formation and release are rapid processes and extremely difficult to capture. Not surprisingly, there are few images in the literature of the exact moment that a conventional OMV is released from a bacterium [6], [31], [32]. It is therefore notable that we were able to visualize the formation of a new O-IMV in *N*. *gonorrhoeae*.

It should also be emphasized that the new O-IMVs and their proportion were analyzed in samples obtained under optimal culture conditions to prevent cell lysis, which could generate membrane recircularization leading to artifactual atypical vesicles. Furthermore, all analyses were carried out in the absence of agents that could alter the cellular integrity, such as antibiotics. This is worthy of mention, since atypical membrane vesicles with a possible resemblance to O-IMVs have been formed in the presence of antibiotics. For example, Kurundurungawa and Beveridge observed that after treatment with gentamicin, *P*. *aeruginosa* PAO1 had an altered cellular appearance and produced a complex type of vesicle incorporating PM and cytoplasmic content [31]. However, the definition of the images provided by this study is too low to distinguish whether the vesicles are equivalent to the O-IMVs spontaneously produced by our test strains [31]. A study of the *A*. *baumannii* strain ATCC19606T by Cryo-electron tomography also provided images of MVs apparently with a PM and OM, whose production increased when suboptimal concentrations of antibiotic were added to the cultures [33]. In a Cryo-TEM study of *Borrelia burgdorferi* the addition of the borreliacidal antibody OspA induced the formation of very large vesicles containing a material very similar to cytoplasm, which may also resemble the O-IMVs described here [34]. These observations suggest that O-IMV production could be induced by bacteria in stressful or adverse situations. This would be of particular importance in pathogenic bacteria, because an antibiotic treatment or the host immune system could stimulate the production of new O-IMVs, with implications for the development of bacterial infection and the appearance of antibiotic resistance.

The presence of DNA in MVs of *N*. *gonorrhoeae*, *P*. *aeruginosa* and *A*. *baumannii* has been described [17], [20], [21]. Furthermore, the transfer of antibiotic resistance plasmids by MVs has been shown in *N*. *gonorrhoeae* and *A*. *baumannii*. None of these studies resolved the question of how the DNA is included in the interior of the vesicles, and to date three possible mechanisms have been proposed [22]. Renelli et al suggest that the DNA present in the medium due to cell lysis can be internalized by a mechanism similar to bacterial transformation [17]. Alternatively, the DNA somehow passes through the periplasm of a protected form and is included in a conventional OMV [31]. This model has never been demonstrated experimentally, although several articles defend its validity [5], [17]. Finally, the third model demonstrated in the Antarctica strain *S*. *vesiculosa* M7^T^ by our group, explains the inclusion of DNA by the formation of more complex vesicles (O-IMVs), which incorporate both PM and cytoplasmic content, and therefore also DNA. Our current work shows that this third model can be extended to other Gram-negative bacteria species, since the O-IMVs formed by the three analyzed strains internalize DNA in a way that protects it from the action of exonucleases. Furthermore, DNA immunolabeling with a specific antibody for dsDNA allowed us to visualize and confirm that the quantified DNA was packaged within the O-IMVs of the pathogenic bacteria. The type of DNA in the vesicles could not be distinguished by immunolabeling, although vesicles of *N*. *gonorrhoeae* have been reported to contain both plasmid and chromosomal DNA [20].

The proteomics of MVs have been reported in *A*. *baumannii* and *P*. *aeruginosa*, but not, as far as we know, in *N*. *gonorrhoeae* strains [9–11]. Thus, the dual aim of our proteomic study was to identify which proteins were exported in the MVs of *N*. *gonorrhoeae* and to determine their subcellular localization. As in *A*. *baumannii* and *P*. *aeruginosa*, the proteomic analysis identified more proteins of the cytoplasm (C) and PM than the periplasm (P) and OM, although in total OM proteins were more abundant than those of the C and PM [9–11]. While OM and P proteins are considered a natural component of Gram-negative bacteria MVs, the presence of C and PM proteins lacks a clear explanation.

Some authors attribute the presence of cellular debris to improper preparation of vesicle samples [1]. Although this is possible, it seems unlikely to have occurred in all the rigorous studies published [7], [8], [12], [16]. Another possible explanation for the presence of cytoplasmic proteins is that some are highly conserved moonlighting proteins, which may have multiple simultaneous locations and perform more than one biological function. Several moonlighting proteins, which exercise their primary function in the cytoplasm, have also been found on the surface of bacteria acting as virulence factors [35], [36]. This would be the case of some of the proteins identified in the proteomic study of *N*. *gonorrhoeae* MVs, such as pyruvate dehydrogenase, enolase, chaperone DnaK and elongation factor Tu [36], [37]. However, based on current knowledge, many of the proteins detected in *N*. *gonorrhoeae* MVs would be localized exclusively in the cytoplasm or PM, including those associated with the ATP synthase complex and ribosomal proteins. Finally, a third explanation is that C and PM proteins were incorporated into the O-IMVs in the vesiculation process.

Another interesting finding in the analysis of the three pathogenic strains was the presence of ATP inside their MVs. To our knowledge, this is the first time that ATP has been described in MVs of Gram-negative bacteria. This is a noteworthy feature since ATP is a unique component of the cell cytoplasm, being considered the universal energy currency of many biological processes, such as active transport, nucleic acid synthesis and movement [38]. As in other components, the presence of ATP in the O-IMVs can be explained by its incorporation during vesiculation, but its functionality remains unclear. However, several of the proteins identified in the proteome of MVs from *N*. *gonorrhoeae*, *P*. *aeruginosa* and *A*. *baumannii* contain ATP binding sites and require the ATP cofactor to function properly, as is the case of enzymes involved in glycolysis or different chaperones; additionally, several subunits of the ATP synthase have been identified.

The detection of ATP in MVs raises various issues. On the one hand, there is the possibility that some of the vesicle proteins maintain their functionality once outside the cell. On the other, the presence of ATP might contribute to the inter- and intraspecies communication function of MVs, since it has recently been postulated that ATP can be used as an intercellular communication signal by eukaryotic and prokaryotic cells [5], [39]. Moreover, Mempin et al have found that Gram-negative bacteria release ATP into the culture medium during growth, although the mechanism remains to be determined [26]. It would be interesting to explore whether O-IMVs are involved in this secretion.

In conclusion, the production of these new O-IMVs, not only by Antarctic bacteria, but also by pathogenic Gram-negative bacteria, is significant, as it implies the existence of another vesiculation model. Besides their novelty, these vesicles offer an explanation for the presence of cytoplasmic and inner membrane components repeatedly described in MVs of Gram-negative bacteria. This assumes particular significance in the case of pathogenic bacteria in which MVs are associated with the transfer of DNA, toxins and other virulence factors. Future work should be addressed to confirming the role of these O-IMVs in pathogenesis and the factors that trigger their formation.

## Supporting Information

S1 Table Proteins identified from *N*. *gonorrhoeae* MVs with at least 2 high confidence peptides. Molecular weight (MW); amino acid length (AA); cluster of orthologous groups (COG).
(XLSX)

S1 Fig DNA gold immunolabeling on Lowicryl HM20 thin sections of HPF-FS isolated MVs from *N*. *gonorrhoeae*. TEM micrographs showing O-IMVs immunolabeled with a monoclonal IgM specific against dsDNA and a secondary goat anti-mouse antibody coupled to 12-nm colloidal gold (black arrows). Bars, 100 nm.
(TIF)
