# Supplementary figures and images for: Outer-Inner Membrane Vesicles Naturally Secreted by Gram-Negative Pathogenic Bacteria
Source: PLoS One. 2015 Jan 12;10(1):e0116896. doi: 10.1371/journal.pone.0116896 (PMC4291224; doi:10.1371/journal.pone.0116896)

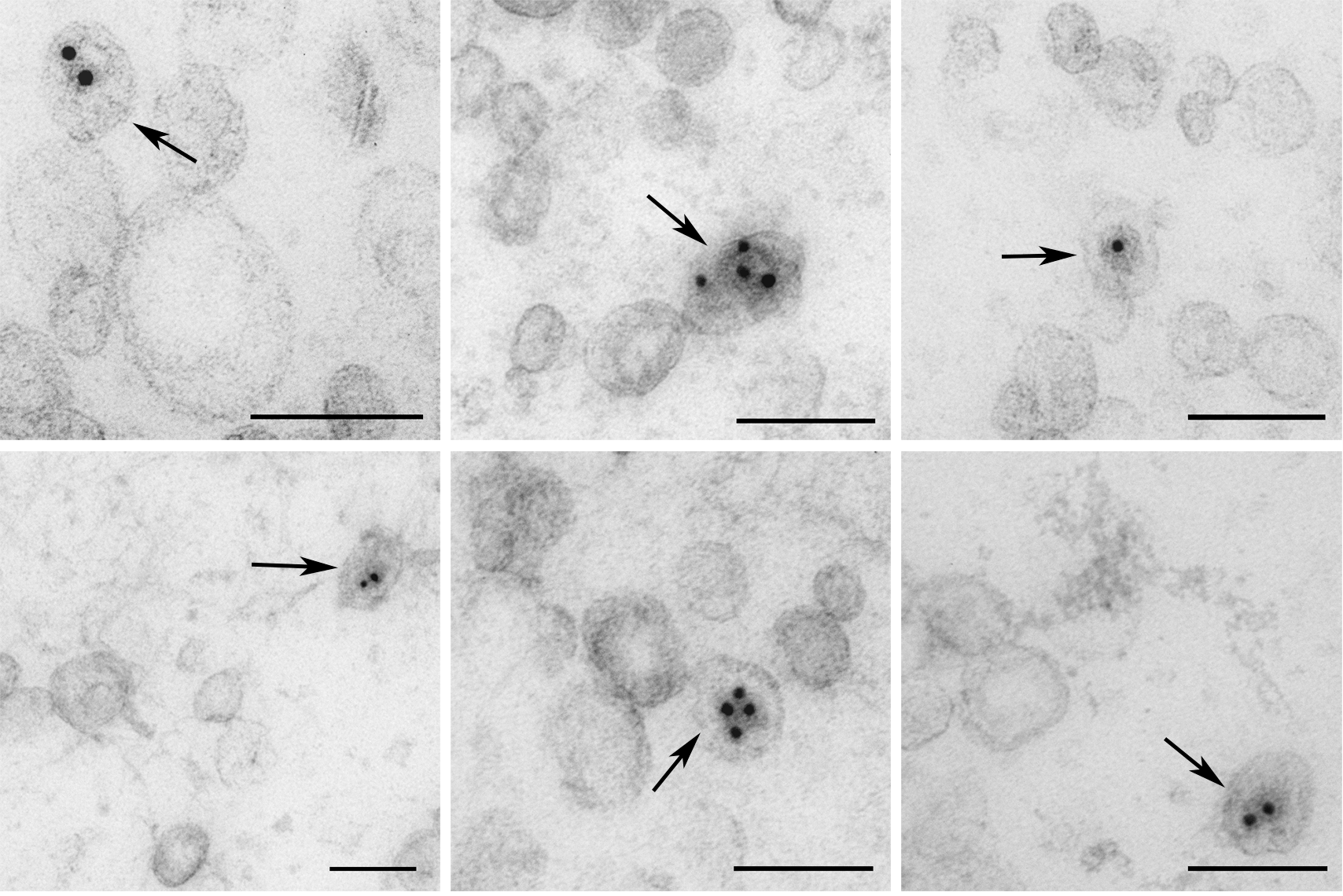

Supplement: S1 Fig — TEM micrographs showing O-IMVs immunolabeled with a monoclonal IgM specific against dsDNA and a secondary goat anti-mouse antibody coupled to 12-nm colloidal gold (black arrows). Bars, 100 nm. (TIF) [file pone.0116896.s002.tif]
